# P02-12 The implementation cost of a walking football exercise program for patients with type 2 diabetes: a case study of SWEET-Football (Portugal)

**DOI:** 10.1093/eurpub/ckac095.031

**Published:** 2022-08-29

**Authors:** Ana Barbosa, João Brito, Pedro Figueiredo, André Seabra, Romeu Mendes

**Affiliations:** 1 EPIUnit, EPIUnit - Instituto de Saúde Pública, Universidade do Porto, Porto, Portugal; 2 ITR, Laboratório para a Investigação Integrativa e Translacional em Saúde Populacional (ITR), Porto, Portugal; 3 Portugal Football School, Portuguese Football Federation, Oeiras, Portugal; 4 Centro de Investigação em Desporto, Educação Física, Exercício e Saúde (CIDEFES), Universidade Lusófona, Lisboa, Portugal; 5 Research Centre in Physical Activity, Health and Leisure (CIAFEL), Faculdade de Desporto, Universidade do Porto, Porto, Portugal; 6 ACES Douro I - Marão e Douro Norte, Administração Regional de Saúde do Norte, Vila Real, Portugal

**Keywords:** Exercise, Sports, Noncommunicable diseases, Economic analysis, Cost analysis

## Abstract

**Background:**

Economic analysis of health interventions is essential to the development and implementation of sustainable health policies, especially in noncommunicable diseases area. Type 2 diabetes (T2D) is one of the most relevant noncommunicable diseases globally. Regular physical activity is an established cornerstone of T2D control, with benefits in glycemic control, cardiovascular risk factors and quality of life. Thus, the current study aimed to assess the cost of a community-based physical activity intervention for patients with T2D.

**Methods:**

We assessed the SWETT-Football program - a community-based walking football exercise program for middle-aged and older male patients with T2D. The program was tested in Portugal through a scientific project (NCT03810846) funded by FIFA (FIFA Research Scholarship 2018). One season of this program consists of three sessions per week (60 minutes per session) during nine months (October to June). For the calculations, we considered a total of 40 patients (two groups of 20). We calculated the direct costs of one season for the host institution: 216 hours of renting a sports hall and hiring human resources (a football coach and a nurse); cardiac stress tests and sports insurance for the participants; sports equipment (balls, cones, vests); vital signs monitoring equipment (blood pressure, heart rate and capillary blood glucose); logistical equipment (disposable and non-disposable); and technical training. In addition, we considered an economic depreciation of five years for sports and electronic materials. Cost analysis dated January 2022.

**Results:**

One season of this program for 40 patients with T2D was estimated to have a total implementation cost of 11,026.51€: 1,225.17€/month; 275.66€/patient; 51.05€/session; 30.63€/patient/month; and 2.55€/patient/session.

**Conclusions:**

A community-based walking football program for patients with T2D has an affordable cost and is feasible for large-scale implementation by local communities with the involvement of football clubs, municipalities and primary health care units, promoting physical activity and contributing to T2D control.

